# Megacolon as a Feature of Suspected Robinow Syndrome

**DOI:** 10.7759/cureus.30663

**Published:** 2022-10-25

**Authors:** Zachary M Waarala, Rijul S Maini, Paul Kowalski, William McMillan, Loro L Kujjo

**Affiliations:** 1 Department of Osteopathic Medicine, Michigan State University College of Osteopathic Medicine, East Lansing, USA; 2 Department of Physiology, Michigan State University College of Osteopathic Medicine, East Lansing, USA; 3 Department of Anatomy, Division of Human Anatomy, Michigan State University College of Osteopathic Medicine, East Lansing, USA

**Keywords:** hirschsprung disease, colon, amyloidosis, adhesions, robinow syndrome, megacolon

## Abstract

This study presents the routine prosection findings of a 73-year-old male cadaver, with the cause of death reported to be hypertension and respiratory failure. Deep thorax and abdomen dissection exposed profound external and internal anatomical abnormalities. Externally, the body exhibited the following: pectus excavatum; short-limbed dwarfism; and abnormalities of the head, face, and external genitalia. Most of these findings suggest that the donor had Robinow syndrome, a rare genetic disorder involving developmental delay and skeletal abnormalities akin to those found in this cadaver. The internal gross anatomical findings included the following: megacolon; hiatal hernia; bilateral inguinal hernias; laterally displaced right kidney with a fibrous adhesion extending from the inferior pole of the kidney to the inguinal canal; atypical branching of the abdominal aorta; superiorly displaced diaphragm; pulmonary hypoplasia; heart right of midline; and curved esophagus. Although determining the exact etiology of megacolon is difficult in a cadaveric specimen, it is important to investigate the physiological changes associated with it. Therefore, the aim of this study was to investigate the space-occupying pathology of megacolon and to discuss a potential connection between megacolon and Robinow syndrome.

## Introduction

Whereas some of the findings in this article were previously presented both as a poster and a podium presentation at the Michigan Osteopathic Association 123rd Annual Spring Scientific Convention (May 19-22, 2022, at the Westin in Southfield Detroit, Michigan, United States), the details herein further explore in depth, the pathophysiology and histopathology underlying the megacolon and other gross pathoanatomical findings in the cadaver presented. Megacolon is defined as acute, toxic, or chronic distension of the large intestine in the absence of mechanical obstruction [1]. Large colons affected by megacolon demonstrate extensive luminal dilation, often greater than 10 cm in diameter (particularly in the cecum), and depending upon the etiology, may show a reduction in ganglion cell and neuronal distribution and/or activity. These anatomic changes lead to a disruption in peristaltic activity, resulting in variable clinical presentations, such as severe abdominal pain, distension, and constipation. Acute megacolon can be idiopathic or occur secondary to surgical intervention and trauma. Toxic megacolon is associated with Chagas disease, C. difficile pseudomembranous colitis, and inflammatory colitis (e.g., ulcerative colitis and Crohn’s disease). Chronic megacolon can be acquired or congenital, most often from Hirschsprung disease, which is the most common congenital neuropathy and is associated with chromosomal disorders [1,2].

The cadaver presented in this article exhibited gross anatomical features of Robinow syndrome and megacolon. This is a unique situation since the database is devoid of any description of the association between the two conditions. Robinow syndrome is a genetic disorder that affects bone development and often manifests as genital deformity or craniofacial abnormalities such as cleft lip/palate [3,4]. It would therefore be interesting to determine if megacolon is also a pathology of Robinow syndrome. Robinow syndrome has been reported in fewer than 200 cases in the literature and confirming an association with megacolon would help guide the treatment and prognosis of this rare condition. In cadaveric dissection, which forgoes clinical presentation and limits diagnostic tests, differentiating between the etiologies of megacolon is difficult. Nonetheless, post-mortem discoveries of megacolon are worthy of discussion in order to better understand the sequelae of physiologic events and associated pathology. That is, although the determination of the exact cause of megacolon is difficult in cadavers, it is easy to discern the gross anatomical changes that occurred secondary to the presence of megacolon. Those gross changes, of course, can be pathologic or associated with histopathological changes.

## Case presentation

Pathoanatomical findings

**Figure 1:**
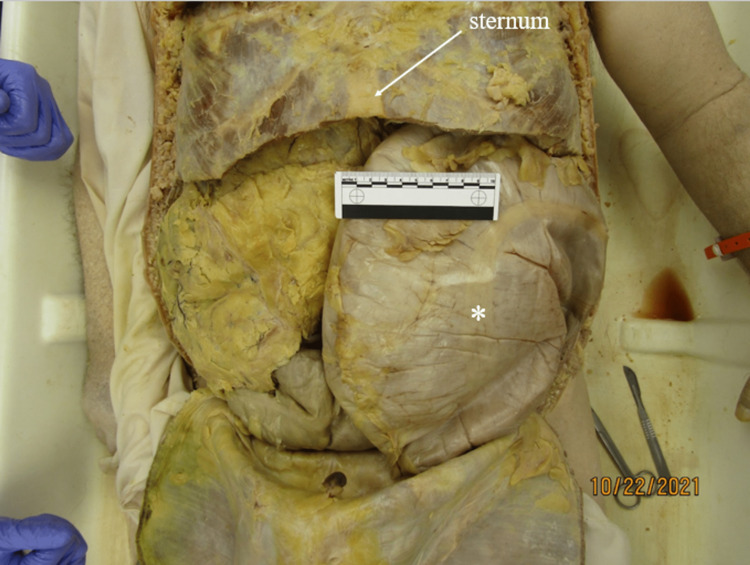
Megacolon in situ Descending colon is marked with an asterisk (*). Ruler in image measures 10cm.

**Figure 2:**
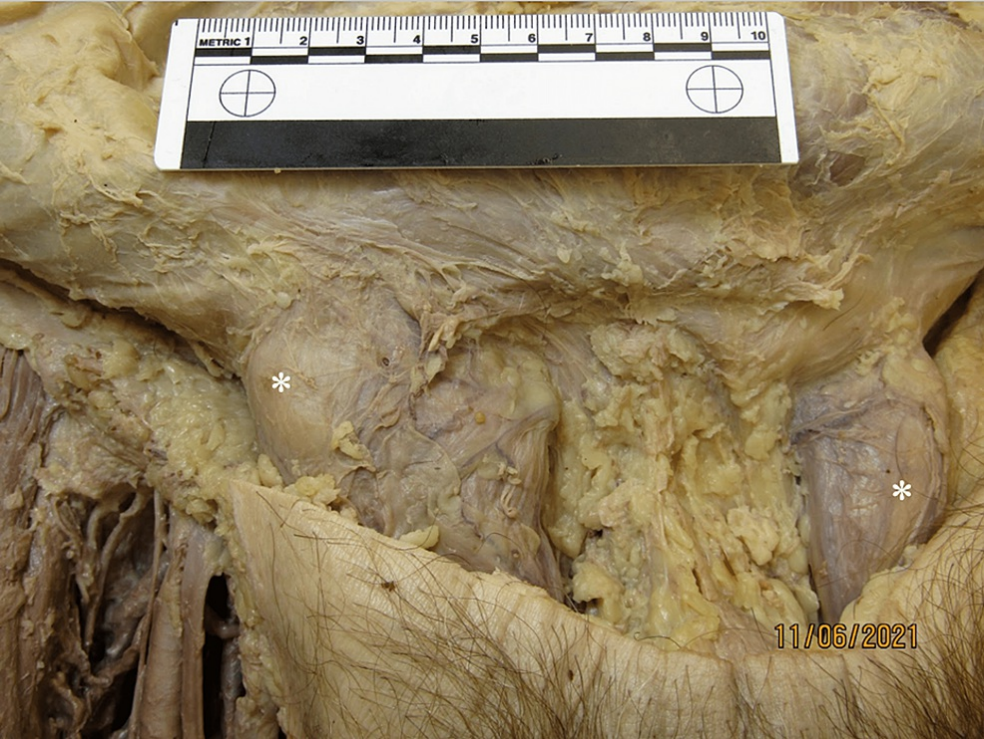
Bilateral inguinal fat Bilateral herniations (*) of abdominal fat. Ruler in image measures 10cm.

**Figure 3:**
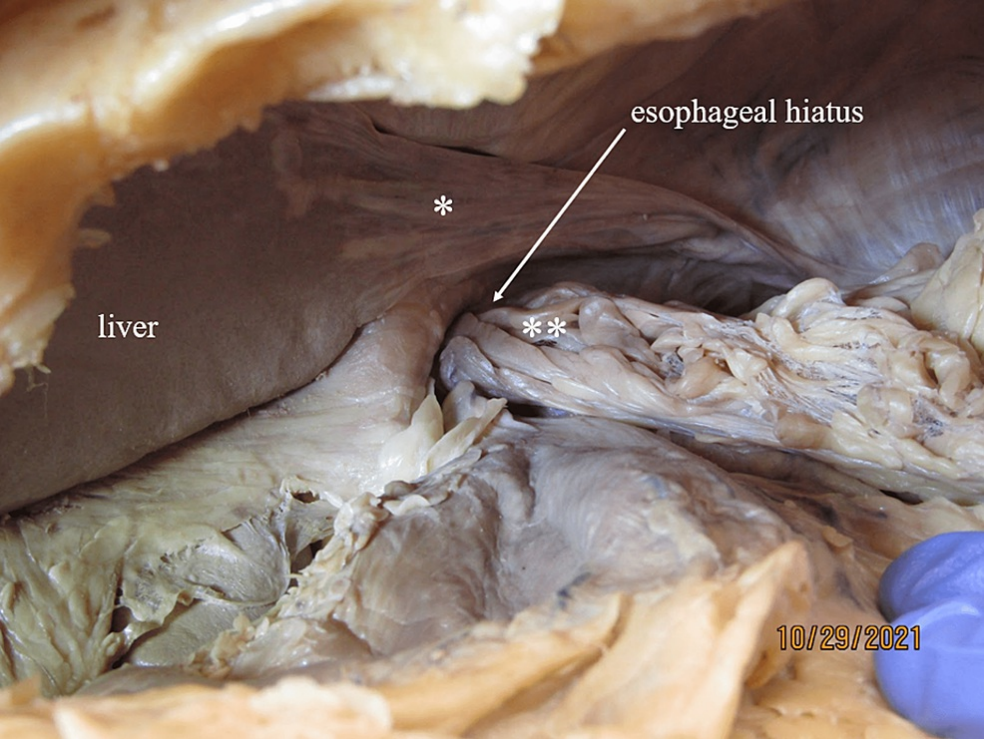
Fibrous band from liver and hiatal hernia Thick fibrous extensions of the left triangular ligament (*) extended from the medial border of the liver and attaches on the posterior abdominal wall deep to the spleen. A portion of omentum (**) had herniated through the esophageal hiatus (arrow), settling inferior to the heart (also see Figure 12).

**Figure 4:**
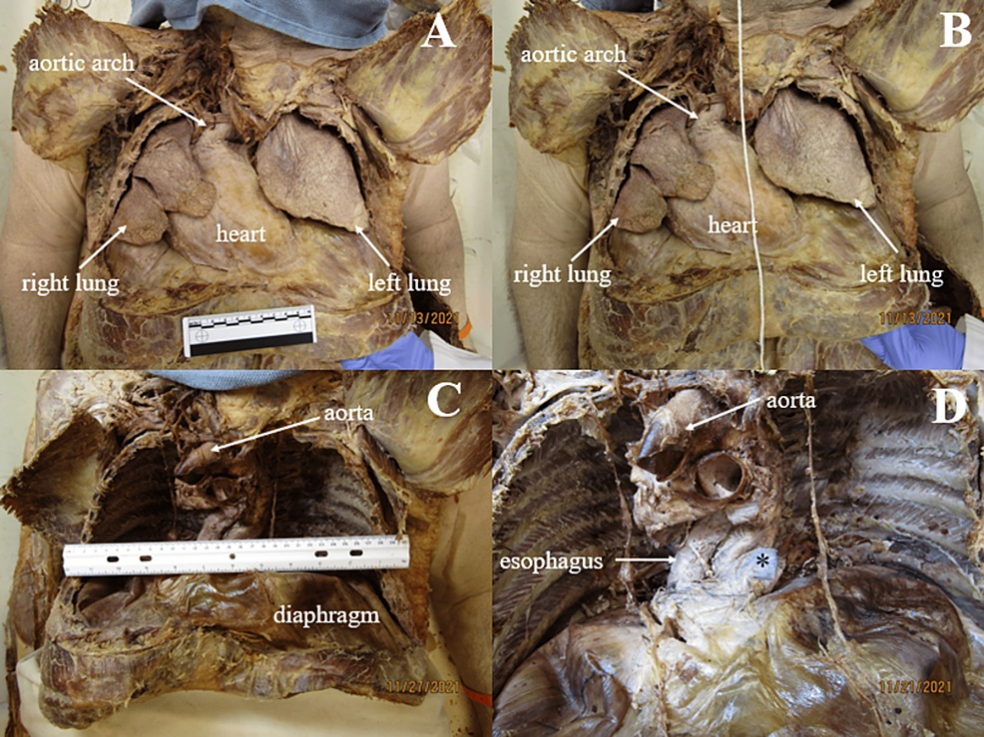
Thoracic abnormalities Panel A: Thoracic organs in situ revealing right displacement of the heart and lungs. Panel B: String was held at approximately midline to show displacement of the heart to the right of midline. Panel C: Narrow thoracic cavities, each measuring approximately 28cm in width. Panel D: Anterior view of thorax. Finger (*) pushed through hiatal hernia sac to show where the hernia was previously located. Esophagus is visibly displaced to the right. Ruler in images measures 10cm.

**Figure 5:**
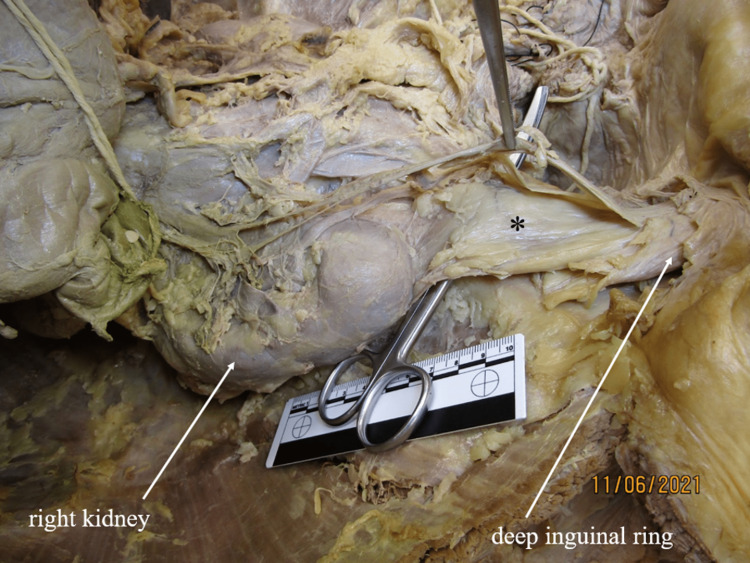
Fibrous band through inguinal canal Fibrous, ligamentous adhesion (*) extending from the inferior pole of the right kidney and passing through the inguinal canal into the scrotum. The testicular artery, vein, and ureter are retracted to show the fibrous surface. Ruler in image measures 10cm.

During routine dissection of a 73-year-old male cadaver, multiple anatomical variations and abnormalities were discovered. The main observations included the following: megacolon (Figure 1); bilateral inguinal fat hernias (Figure 2); hiatal hernia (Figure 3); superior displacement of the diaphragm, heart, and lungs (Figure 4); vertebral displacement; and the presence of multiple fibrous adhesions throughout the abdomen. A strap of fibrous band extended from the inferior pole of the right kidney through the inguinal canal and terminated in the scrotum (Figure 5). Another fibrous band was found alongside the inferior aspect of the diaphragm and abdominal wall, posterior to the liver (Figure 3). Weights of the heart, lungs, and liver were within normal ranges, (although at the higher end) for adult males (Table 1).

**Table 1:** Post-mortem organ weights Weight of heart, lungs, and liver in the cadaveric donor compared with normal values for men. Note that the figures are at the higher end for adult males. [5,6].

Organ	Weight (grams)	Normal Weight Range for Adult Men (grams)
Heart	545	188-575
Right lung	590	185-967
Left lung	630	186-885
Liver	1485	838-2584

**Figure 6:**
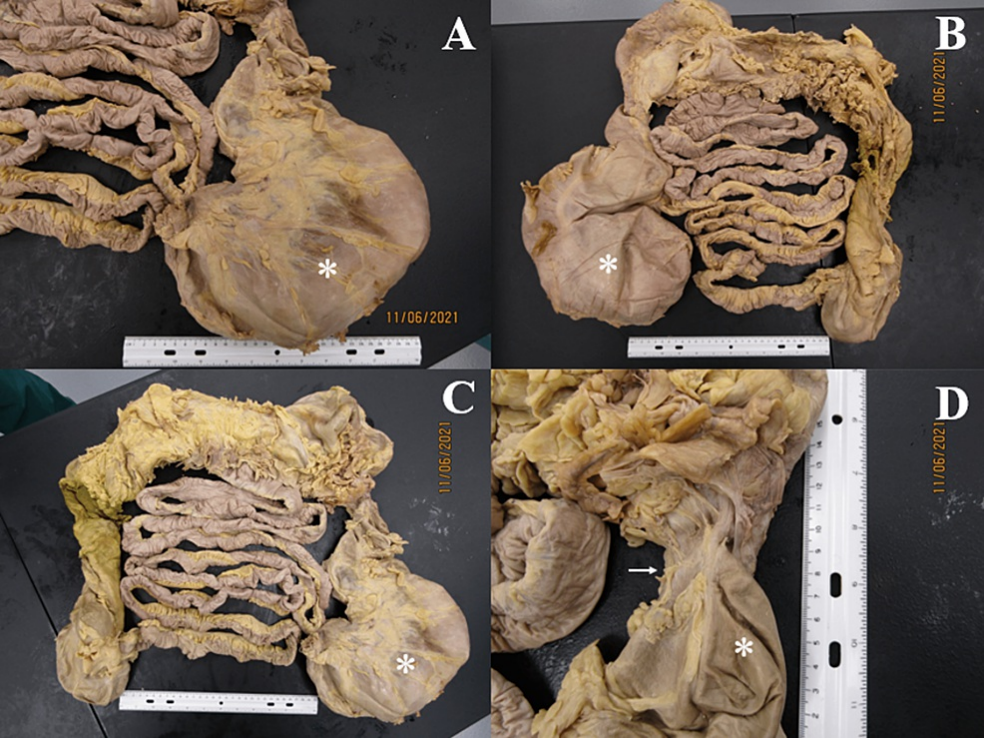
Small and large bowel ex vivo Panel A: Anterior view and Panel B: Posterior view of small and large intestine removed from cadaver and rearranged ex-vivo to show extensive megacolon (*), particularly of the descending segment. Constrictions of colon can be seen at the hepatic and splenic flexures. The small intestine appears relatively normal. Panel C: Megacolon of the descending segment measures approximately 23cm at its largest diameter. Panel D: Splenic flexure shows extensive fibrous adhesions (arrow) at the constricted portion of the descending colon along a length of 6cm. Ruler in images measures 30cm.

The colon was dilated, particularly the descending segment, which featured a maximal dilation measuring approximately 23 cm. Comparatively, the enlargements of the cecum, ascending and transverse colon were not as remarkable. The hepatic and splenic flexures were markedly narrowed (Figure 6). In contrast, the small bowel, stomach, and esophagus were of normal sizes.

**Figure 7:**
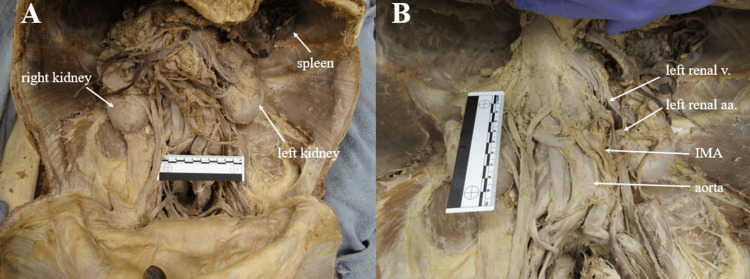
Abdominal abnormalities Panel A: Anterior view of abdomen. Right kidney was found rotated externally, exposing the hilum anteriorly while left kidney was in relatively normal position. Panel B: Anterior view of abdomen demonstrating proximal origin of the inferior mesenteric artery and accessory renal arteries. The ruler in the images measures 10cm. IMA= inferior mesenteric artery; v= vein; aa= arteries

The gross anatomic sequelae of megacolon were easy to notice in this cadaver. For example, it was evident that abdominal and thoracic organs had all been shifted to some degree by mass displacements. The diaphragm, heart, and lungs had all been shifted superiorly (Figure 4). Portion of the greater omentum had herniated through the esophageal hiatus into a space inferior to the heart (Figure 3 and Figure 4). The right kidney was rotated, exposing the hilum anteriorly (Figure 7). In addition to the visceral changes, alterations in vascular architecture were noted in the abdomen. The inferior mesenteric artery branched from the abdominal aorta more proximally than normal and accessory renal arteries were found bilaterally (Figure 7).

This plethora of internal variations and abnormalities prompted to note external features as well, with the following being most prominent: short stature of approximately 5’1” with relatively short limbs; pectus deformity; webbed penis; wide-set eyes; enlarged tongue; and anteverted nares.

Histopathological findings

**Figure 8:**
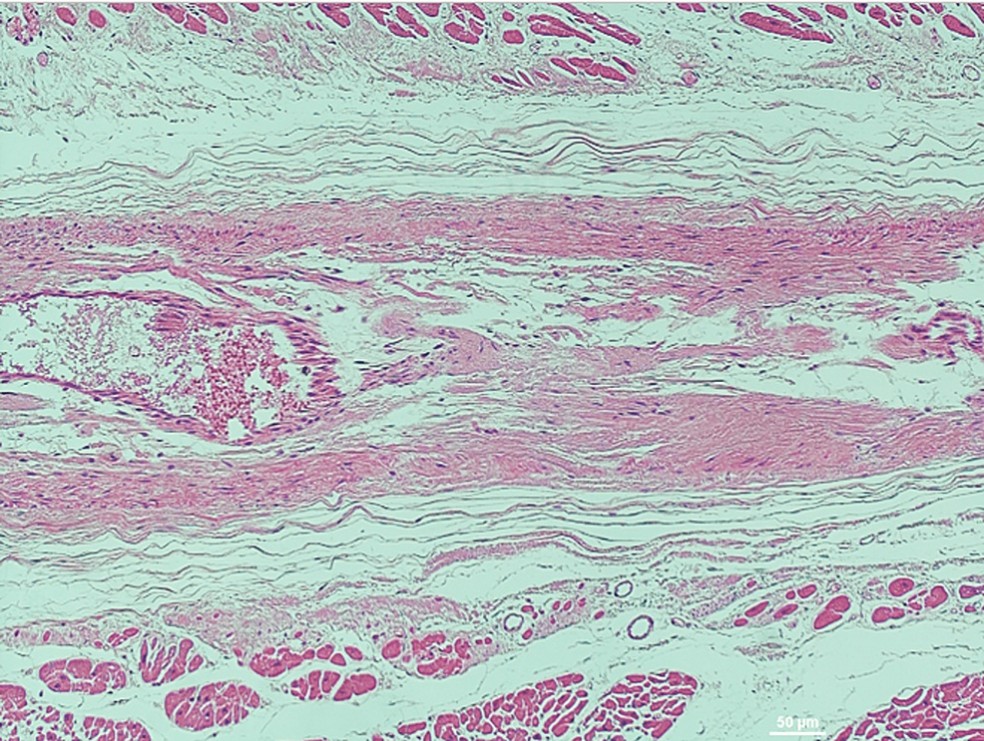
H&E stain of left ventricle Left ventricle at 10X magnification showing diffuse atrophy and dropout of muscle fibers and ischemic change. Diagnosis: Likely left ventricular myocardial fibrosis. Stain: Hematoxylin & Eosin.

**Figure 9:**
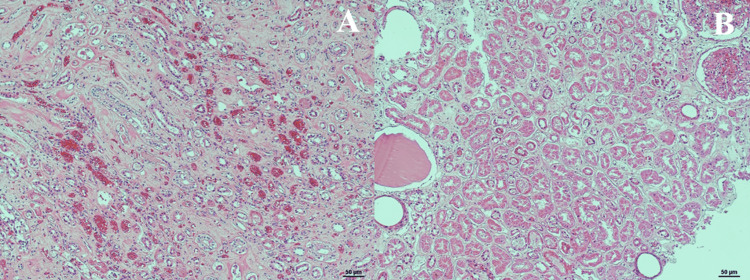
H&E stain of left and right kidneys Panel A: Left kidney at 10X showing tubular dropout and inflammatory cells throughout. Panel B: Right kidney at 10X showing tubular dropout, inflammatory cells, and some tubules containing eosinophilic hyaline material. Stain: Hematoxylin & Eosin.

**Figure 10:**
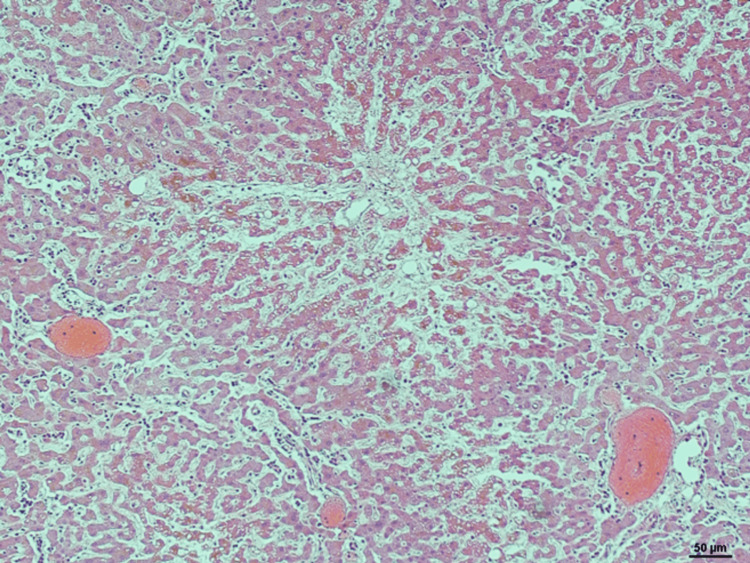
H&E stain of liver Liver at 10X showing scattered inflammatory cells, fibrosis, and triaditis. Stain: Hematoxylin & Eosin.

**Figure 11:**
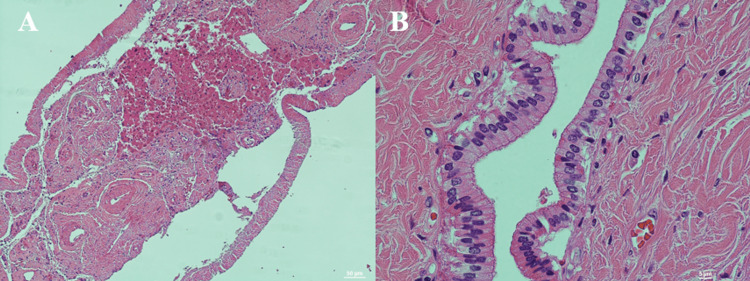
H&E stain of fibrous band from liver Sections from fibrous adhesion extending from the medial tip of the liver to the posterior body wall. Panel A: 10X image shows vasculature, nerve bundles, and ductal structures, as well as scattered inflammatory cells. Panel B: 60X image shows one of the ductal structures lined with columnar epithelium. No specific function could be attributed to this tissue and its structures. Stain: Hematoxylin & Eosin.

Because of the numerous adhesions in the cadaver, the authors wondered if there might have been some involvement of an underlying connective tissue disorder. Sections from various organs stained with H&E and Congo Red were negative for amyloidosis which could have contributed to the organ abnormalities and fibrous pathology. Of note, sections from the left ventricle showed evidence of chronic myocardial fibrosis and muscle atrophy (Figure 8). Histology of the kidneys showed diffuse inflammation of both the right and left kidney with glomerular and tubular dropout. Some tubules of the right kidney contained eosinophilic hyaline material, so-called thyroidization that indicates nephropathy (Figure 9). These kidney changes are likely explained by the history of hypertension and diabetes mellitus. Similarly, throughout the liver, inflammatory cells were scattered alongside fibrosis and triaditis (Figure 10). The thick fibrous ligament extending from the liver (Figure 3) exhibited atypical histology. Sections from the fibrous ligament showed presence of vasculature, nerve bundles, ductal architecture, and columnar epithelium, suggesting that this fibrous ligament is potentially remnant of embryologic origin (Figure 11). The remaining sections exhibited no histopathological changes in tissue ultrastructure.

## Discussion

The extensive anatomical variations and abnormalities on this cadaver could not be explained by the limited medical history provided upon body donation, which included hypertension, hyperlipidemia, type 2 diabetes, GERD, seizures, and repeated falls. Further investigation into the medical history and communication with the family confirmed a history of developmental delay since birth. Interestingly, the family also reported episodes of severe constipation for approximately 20 years before death. However, no specific syndromic diagnosis is listed that would explain the thoraco-abdominal and external findings.

It is clear that this individual suffered severe megacolon. It is reasonable to believe that the condition was long-standing because of the chronic force required to displace viscera of the abdomen and thorax in such a profound manner as was discovered during prosection. Another indication that this case of megacolon is chronic rather than acute or toxic is supported by the 20-year history of constipation prior to death.

Chronic megacolon can be congenital, acquired, or idiopathic [1]. The strictures at the splenic flexure are suggestive of Hirschsprung disease. Diagnosis of Hirschsprung disease requires histologic confirmation of the lack of ganglion cells in the affected segment [7], but due to the rapid deterioration of colonic tissue post-mortem, such a diagnostic test was not possible in this case. If the chronic megacolon was in fact secondary to Hirschsprung disease, the distended colon is unlikely to have been corrected surgically. The first pull-through procedure to correct Hirschsprung disease was described by Orvar Swenson in 1948 [8]. The family reported that in infancy, the donor had “extensive bowel surgery” including partial bowel resection at the Mayo Clinic. Because of the timeline, it is possible that these described surgeries are referring to unpublished preliminary attempts for a surgical correction of congenital megacolon.

Modern-day surgical treatments for congenital Hirschsprung disease include low anterior rectal resection and terminal ileostomy, pull-out anterior rectal resection and terminal ileostomy, and low anterior resection. Post-operative course in these cases is most often uncomplicated. After resection, patients either undergo fecal diversion such as colostomy or intestinal continuity restoration such as subtotal colectomy. Possible complications include those typical of any surgery manipulating the bowel, including hypoproteinemia, anemia, obstruction, abdominal effusion, and infection of the surgical incision [9].

Diagnostic capabilities in cadaveric study are extremely limited; a full history and physical examination are impossible to obtain and neither laboratory nor genetic testing is feasible. As such, all attempts of diagnosis using the cadaver are based on anatomic changes that are appreciable post-mortem as well as limited medical history, augmented by information reported by the family.

Therefore, the authors relied on anatomical cues for genetic pathology to support the hypothesis that the megacolon was indeed congenital. These included the fetal facial features, short stature, accessory renal arteries, genital deformity, heart defects, and the ligamentous structure extending from the liver, showing embryologic features.

With these clues supporting genetic and/or embryologic pathology, Robinow syndrome becomes the most likely diagnosis given the close alignment of symptoms resulting from that disease process and the gross and microscopic findings discovered on this cadaver, namely, short stature, fetal facial features, genital deformity grossly, and left ventricular myocardial fibrosis. There is also a history of developmental delay, which is typically present in cases of Robinow syndrome. Robinow syndrome can result from a variety of gene mutations, and can be autosomal recessive, from a loss of function mutation in the ROR2 gene, or autosomal dominant, from a mutation either to WNT5A or to DVL1 [5,10].

## Conclusions

During routine prosection of a 73-year-old male cadaver, the authors discovered a number of anatomical anomalies that could not be explained by the reported medical history, most notable of which being extensive distension of the descending colon. The anatomical nature of the megacolon and the report from the family indicating constipation 20 years prior to death pointed to a chronic rather than an acute process. The additional internal anatomical variations of accessory renal arteries and atypical fibrous adhesions suggested an underlying congenital cause for these developmental anomalies. These unique external findings narrow that congenital cause to the diagnosis of Robinow syndrome. The nature of cadaveric dissection limits diagnostics, so laboratory and genetic testing were not possible to confirm the diagnosis. Regardless, this case of megacolon in a cadaver illustrates the profound space-occupying effects of megacolon, in that almost every abdominal and thoracic organ was affected in some way due to the sheer size of the defect and consequential mass effect. This highlights the importance of early detection and treatment of megacolon. As for its relation to Robinow syndrome, which is suspected in this cadaver, more genetic research and data would be required to properly correlate megacolon as a feature of Robinow syndrome.
